# Gleanings from the French

**Published:** 1875-01-01

**Authors:** 


### GLEANINGS FROM THE FRENCH.

*Translated by Fred. J. Huse, M.D.*

THE use of Esmarch's Elastic Bandage as a means of obtaining local anæsthesia, formed the subject of a communication by Dr. Chauvel to a recent meeting of the Surgical Society of Paris, and is reported in *La France Medicale* of Nov. 21st, 1874.

In order to eliminate every cause of error, Dr. Chauvel first obtained the measure of the normal sensibility of each patient; then, applying the elastic compression, which he maintained during a certain space of time, he proceeds in accordance with a fixed method to determine the condition of sensibility. Fifteen observations were made, twelve upon the lower extremity, three upon the upper extremity. Only one of the subjects upon which these experiments were made (none of them were subjected to an operation), proved refractory; all the others exhibited a diminution of sensibility. "Anæsthesia never appears immediately, but is developed after from five to twenty minutes. Insensibility is obtained more rapidly in the upper extremity than in the lower extremity, while the degree of constriction of the member by the bandage, and especially by the rubber tube, has a marked influence upon the rate of the development, and upon the degree of the local anæsthesia."

Insensibility first appears in the parts most remote from the trunk, and slowly proceeds towards the centre; it is also in these same parts that it is manifest in the highest degree. In short, in every case, except the one where sensibility seemed to remain perfectly normal, there was an evident, but almost always incomplete, anæsthesia.

While susceptibility to pain is the most rapidly effaced, and the absence of pain is the most noticeable phenomenon, the sense of touch persists throughout, and Dr. Chauvel questions whether the absence of painful sensation would be sufficient to allow of more serious operations than those which he has performed.

Two of these were upon in-growing nails. In the first case the surgeon stripped off a portion of the nail, three lines in breadth, yet the patient affirmed that the suffering was slight. In the second, the remains of the nail were torn away without causing any very severe pain, and the patient easily supported, for more than ten minutes, the application of Vienna paste to the matrix of the nail.

Upon a soldier suffering from irregular pains, probably those of sciatica, the surgeon made cauterizing punctures, which caused very intense pain upon the thigh, less intense pain upon the leg; it was only at the tip of the great toe that the application of the hot iron, made with a certain degree of moderation, failed to produce any pain.

While these facts are by no means conclusive regarding the utility of this method of producing local anæsthesia, it is believed that additional investigation may develop practical results.

ANÆSTHESIA, by simultaneous use of chloral and chloroform, has been lately suggested by Dr. Fornet, to the Surgical Society of Paris, as likely to replace the combined use of opium and chloroform recommended by Claude Bernard, and practiced by many surgeons. One of the cases cited is that of a little girl, five years of age, who had symptoms of stone in the bladder. Thirty grains of chloral were administered, and during the sleep that followed she was caused to inhale chloroform after which the bladder was examined and a deposit of mucus withdrawn, containing gravel.

Another case is that of a vigorous man of twenty-five years, upon whom the administration of chloroform had already failed, suffering from abscess of the anus, with exceedingly painful contraction of the sphincter. Seventy-five grains of chloral were given and in an hour and a half the patient sunk into a quiet sleep. Chloroform was then administered, and the abscess of the rectum was opened without rousing the patient.

According to *La France Medicale*, the ideas which have been formulated by Dr. Fornet are at least worthy of careful consideration. He proves that there is an enormous difference between the administration of chloroform during waking and during sleep. When the patient is awake a large quantity of chloroform is required, and there then exist the dangers of intoxication. Moreover, the patient is excited by the fear of becoming insensible, whence there is danger of nervous sideration. But while he affirms that his method of procedure affords none of these dangers, the two cases he has published are by no means sufficiently convincing.

Drs. Dolbeau, Guyon, and Demarquay, have called attention to some of the unpleasant results which may follow the use of chloroform after doses of chloral, administered by patients to themselves, without the knowledge of the surgeon. A lady under the treatment of the distinguished surgeon of Beaujon Hospital, for an exceedingly painful anal fissure, had been accustomed to take large doses of chloral to alleviate her distress. Upon the morning fixed for the proposed operation by forcible dilatation, Dr. Dolbeau found the patient sleeping, and, being unaware that it was the result of a large dose of chloral taken during the night, she was caused to inhale chloroform and the operation was performed without disturbing the patient. Though only a small amount of chloroform had been found necessary, Dr. Dolbeau was obliged to spend several hours in rousing her from her sleep; when thoroughly shaken she would speak, but as soon as the conversation ceased she would again fall asleep, and the skin would become cold and moist.

Last year Dr. Dolbeau performed resection of the inferior maxillary of an English officer, for a very painful epithelioma. The patient passed readily under the influence of chloroform, and after completion of the operation was easily aroused; but there was a tendency to sleep and coldness which was not overcome until the following day. This patient had taken, during the night preceding the operation, over one hundred and fifty grains of chloral.

Another patient with affection of the rectum had taken more than two hundred grains of chloral the night previous to being operated upon. He continued to manifest a very great tendency to coldness, and only survived forty-eight hours.

Until this association of chloral and chloroform has been tested by further experimentation, it would appear to be advisable, according to the opinion of these gentlemen, to reserve it for certain particular cases, such as those complicated with some nervous disorder, for example.

